# Dectin-1 signaling inhibits osteoclastogenesis via IL-33-induced inhibition of NFATc1

**DOI:** 10.18632/oncotarget.18411

**Published:** 2017-06-08

**Authors:** Xiaoqing Zhu, Yinghua Zhao, Yuxue Jiang, Tianxue Qin, Jintong Chen, Xiao Chu, Qing Yi, Sujun Gao, Siqing Wang

**Affiliations:** ^1^ Department of Hematology, The First Hospital of Jilin University, Changchun 130061, China; ^2^ Department of Cancer Immunology, Institute of Translational Medicine, The First Hospital of Jilin University, Changchun 130061, China; ^3^ Department of Cancer Biology, Lerner Research Institute, Cleveland Clinic, Cleveland, Ohio 44195, USA; ^4^ Department of Hematology, Ningbo Hangzhou Bay Hospital, Ningbo 315336, China

**Keywords:** osteoclast, dectin-1, NFATc1, IL-33, multiple myeloma

## Abstract

Abnormal osteoclast activation contributes to osteolytic bone diseases (OBDs). It was reported that curdlan, an agonist of dectin-1, inhibits osteoclastogenesis. However, the underlying mechanisms are not fully elucidated. In this study, we found that curdlan potently inhibited RANKL-induced osteoclast differentiation and the resultant bone resorption. Curdlan inhibited the expression of nuclear factor of activated T-cells, cytoplasmic 1 (NFATc1), the key transcriptional factor for osteoclastogenesis. Notably, dectin-1 activation increased the expression of MafB, an inhibitor of NFATc1, and IL-33 in osteoclast precursors. Mechanistic studies revealed that IL-33 enhanced the expression of MafB in osteoclast precursors and inhibited osteoclast precursors to differentiate into mature osteoclasts. Furthermore, blocking ST2, the IL-33 receptor, partially abrogated curdlan-induced inhibition of NFATc1 expression and osteoclast differentiation. Thus, our study has provided new insights into the mechanisms of dectin-1-induced inhibition of osteoclastogenesis and may provide new targets for the therapy of OBDs.

## INTRODUCTION

Osteolytic bone diseases (OBDs) are a common complication in rheumatoid arthritis [1], osteoporosis [1] and Paget’s disease [2], as well as in malignancies, such as multiple myeloma (MM) [3]. OBDs can adversely affect the quality of life and survival of patients due to severe bone pain, pathological fractures and hypercalcemia [1, 4]. Bisphosphonates are widely used in the treatment of OBDs [5–7]. New therapeutic reagents have been reported to treat OBDs [8]. However, current therapies rarely halt the progression of OBDs. OBDs are caused mainly by abnormal osteoclast activation and osteoblast inhibition [9, 10]. Therefore, further investigation of new strategies to inhibit the formation and function of osteoclasts will be important for the therapy of OBDs.

Osteoclasts are the only cells known to cause bone resorption [11]. Osteoclasts can be generated *in vitro* from primary macrophages (osteoclast precursors) in the presence of RANKL and M-CSF, two vital factors in promoting osteoclast differentiation and maturation [12]. NFATc1 is the key transcription factor for RANKL-induced osteoclast differentiation [12, 13]. NFATc1 increases the expression of Dcstamp, ATP6V0D2 and Tks5, the key factors in osteoclast cell-cell fusion; and NFATc1 induces the expression of CLC-7, Cathepsin K (Ctsk) and TRAP, which are responsible for osteoclast-induced bone resorption. MafB, IRF8 and BCL6 are inhibitors of NFATc1expression.

Dectin-1, a C-type lectin receptor (CLR), is expressed mainly by macrophages, monocytes and neutrophils [14]. β-1-3-glucans are agonist ligands of dectin-1 and present on the cell wall of various fungal pathogens, such as *Candida albicans* [15–17]. Previous studies indicate that the dectin-1 agonist curdlan suppresses RANKL-induced osteoclastogenesis [18]. However, the mechanisms underlying its anti-osteoclastogenic effects need to be further elucidated.

In this study, we showed that dectin-1 potently inhibited the differentiation and bone resorption of osteoclasts induced by RANKL plus M-CSF. Dectin-1 activation by curdlan in osteoclast precursors increased MafB expression and decreased NFATc1 expression, suggesting that dectin-1 inhibits NFATc1 through the stimulation of MafB. Interestingly, dectin-1 increased IL-33 expression in osteoclast precursors. Mechanistic studies revealed that IL-33 also increased MafB expression and decreased NFATc1 expression in osteoclast precursors and inhibited osteoclast precursors to differentiate into mature osteoclasts. Furthermore, blocking ST2 (IL-33 receptor) partially abrogated curdlan-induced inhibition of NFATc1 expression and osteoclast differentiation. Thus, our study has provided new insights into the mechanisms of dectin-1-induced inhibition of osteoclastogenesis and may provide new targets for the therapy of OBDs.

## RESULTS

### Dectin-1 activation inhibits osteoclastogenesis *in vitro*

**Figure 1:**
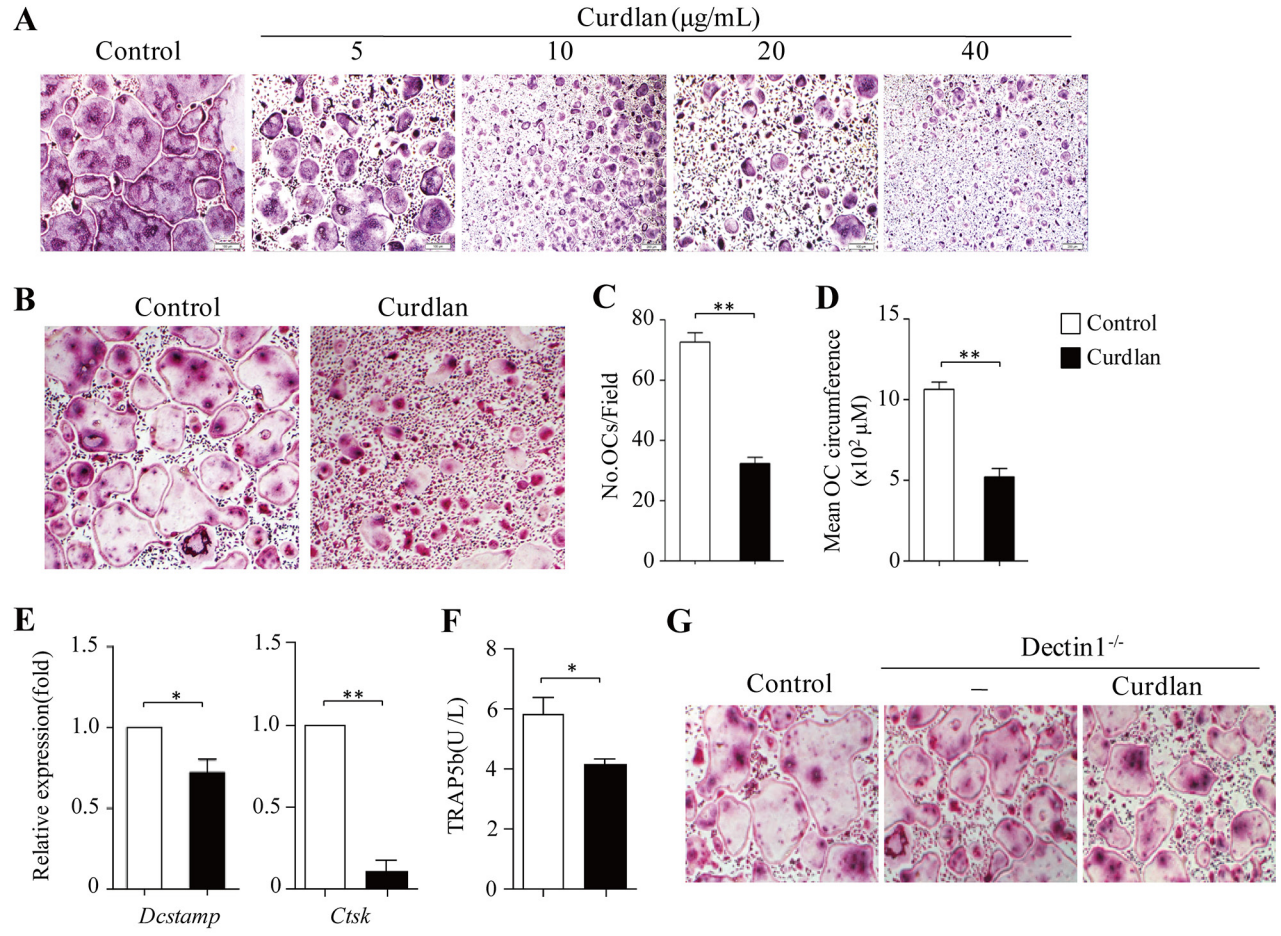
Dectin-1 signaling inhibits osteoclastogenesis **(A-F)** Mouse bone marrow cells (BMCs) were cultured with M-CSF for 2 days and with MCSF plus RANKL for 3 days. **(A)** curdlan at the indicated dosages was added at day 2. Cultures without addition of curdlan were used as controls. Cultures were stained for TRAP^+^ cells. TRAP^+^ cells with more than three nuclei were counted as osteoclasts (OCs). **(B-F)** Cells were treated with or without (control) curdlan (10 μg/mL unless otherwise indicated) at day 2. **(B-D)** At day 5, cells were stained for TRAP^+^ cells. **(B)** Representative TRAP^+^ cells from the cultures. **(C and D)** The cell number **(C)** and mean cell circumference **(D)** of OCs as obtained in **(B)**. (**E and F**) At day 5, cells and culture supernatants were collected. **(E)** qPCR analysis of *Dcstamp* and *Ctsk* in cells. Expression was normalized to *Gapdh* and set at 1 in control cells. **(F)** ELISA assessed TRAP5b secretion in the cultures. **(G)** BMCs from dectin1-deficiency (Dectin1^−/−^) were cultured for OCs with or without (−) curdlan treatment. OCs generated from wild-type (WT) mice were used as control. Data are representative of three independent experiments **(B,G)** or presented as mean ± SD of at least three independent experiments **(C-F)**. *P < 0.05; **P < 0.01.

To examine the effects of dectin-1 signaling on osteoclastogenesis, we cultured bone marrow cells (BMCs) with RANKL plus M-CSF in the presence or absence of a selective dectin-1 agonist Curdlan. Curdlan treatment inhibited RANKL-induced osteoclast formation by decreasing the number and size of TRAP^+^ multinucleated (> 3 nuclei) osteoclasts in a dose-dependent manner (Figure 1A–1D). Concomitant to the inhibition of osteoclast formation, mRNA expression of *Dcstamp* for osteoclast fusion and *Ctsk* for bone resorption and the protein levels of bone resorption-related gene TRAP5b in the culture supernatants were also decreased by curdlan treatment (Figure 1E, 1F).

To explore the function of dectin-1 in curdlan-induced inhibition of osteoclast formation, we generated osteoclasts from dectin-1 knockout (dectin-1^−/−^) mice with or without addition of curdlan. As shown in Figure 1G, curdlan treatment failed to inhibit dectin-1^−/−^ osteoclast formation as compared to the untreated controls.

To assess the effects of curdlan on osteoclast bone resorption, we performed resorption pit formation assay. As compared to untreated controls, curdlan treatment remarkably diminished RANKL-induced osteoclast bone resorption (Figure 4F, 4G). Together, these results demonstrated that dectin-1 activation in osteoclast precursors inhibits osteoclast differentiation and bone resorptive function.

### Dectin-1 signaling inhibits NFATc1 in osteoclast precursors

**Figure 2:**
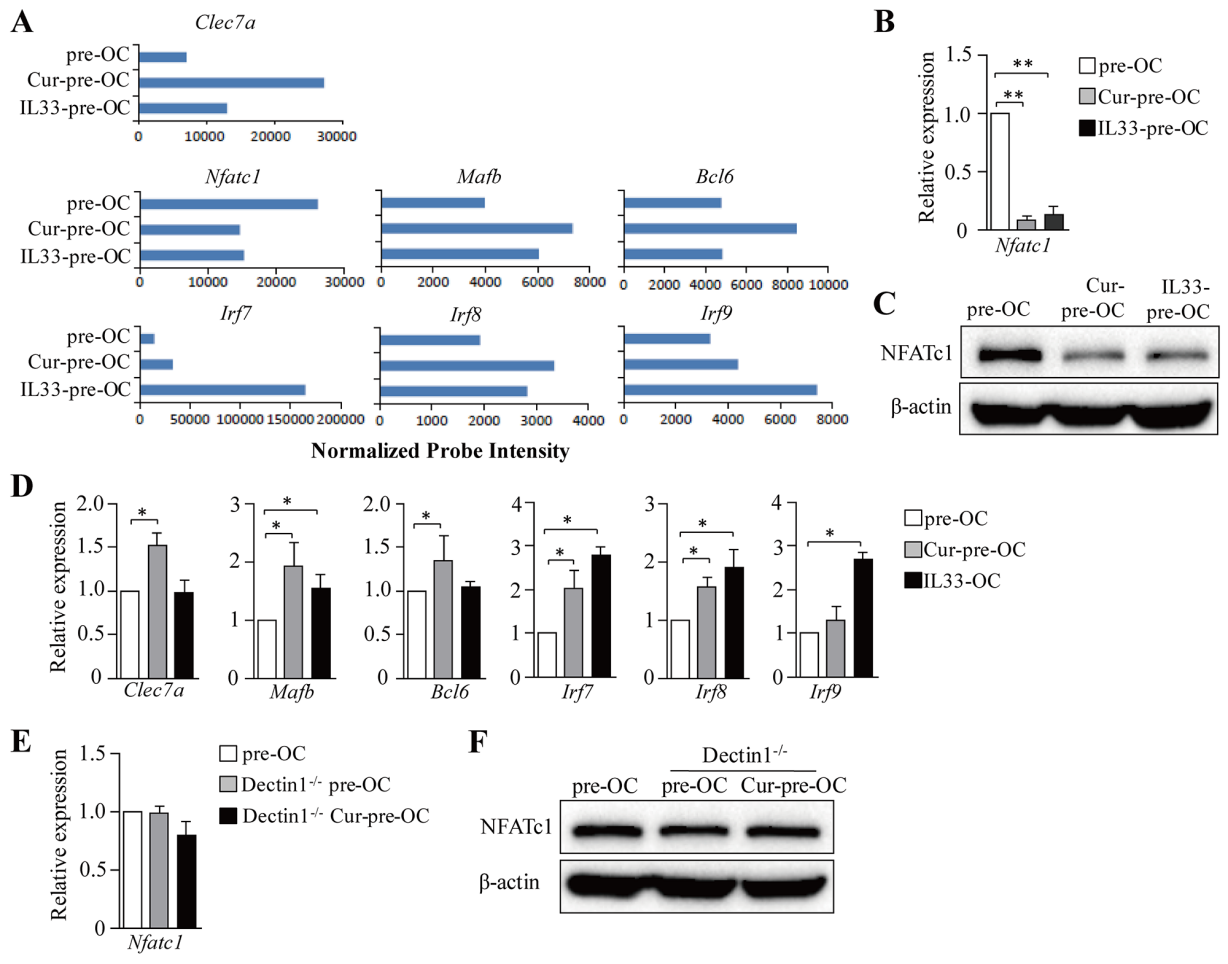
Dectin-1 signaling inhibits NFATc1 in OC progenitors **(A-D)** Mouse BMCs were cultured with M-CSF for 2 days and with M-CSF plus RANKL in the presence of curdlan (Cur-pre-OCs) or IL-33 (IL33-pre-OCs) for another 2 days. Cells without treatment of curdlan or IL-33 were used as controls (pre-OC). **(A)** Cells were analyzed by microarrays. Shown are gene expression levels of the indicated genes extracted from microarray data. **(B and C)** qPCR **(B)** and Western blot **(C)** assessed the expression of NFATc1 in cells. **(D)** qPCR assessed the expression of the indicated genes in cells. **(E and F)** BMCs from Dectin1^−/−^ mice were cultured for OCs and treated with curdlan as shown in **(A)**. OCs generated from WT and Dectin1^−/−^ mice without curdlan treatment were used as controls. qPCR **(E)** and Western blot **(F)** assessed the expression of NFATc1. Results shown are the mean + SD of three independent experiments **(B,D,E)** or representative of at least two independent experiments **(C,F)**.*P < 0.05; **P < 0.01.

To explore the molecular mechanisms of dectin-1-induced inhibition of osteoclast differentiation, we performed gene expression profiling (GEP) analyses in osteoclast precursors with (Cur-pre-OC) or without (pre-OC) curdlan treatment. We found that Cur-pre-OCs expressed lower levels of *Nfatc1*, the master transcription factor for osteoclast differentiation [12], than pre-OCs (Figure 2A); whereas the transcription factors *Mafb*, *Bcl6*, *Irf7*, *Irf8* and *Irf9* were upregulated in Cur-pre-OCs compared to pre-OCs (Figure 2A). Interestingly, Cur-pre-OCs expressed higher levels of *Clec7a* (the gene for dectin-1) as compared to pre-OCs (Figure 2A). The decrease of NFATc1 in Cur-pre-OCs compared to pre-OCs was confirmed by quantitative real-time PCR (qPCR) (Figure 2B) and Western-blot analysis (Figure 2C). The up-regulation of *Mafb*, *Bcl6*, *Irf7* and *Irf8* in Cur-pre-OCs compared to pre-OCs was confirmed by qPCR (Figure 2D).

To further explore the contribution of dectin-1 in curdlan-induced inhibition of NFATc1 expression, dectin-1^−/−^ mice were used. Curdlan treatment failed to inhibit NFATc1 expression in dectin-1^−/−^ Cur-pre-OCs compared to WT or dectin-1^−/−^ pre-OCs (Figure 2E, 2F). MafB, IRF8 and Bcl-6 were reported to be inhibitors of NFATc1 [13]. Thus, our data suggested that dectin-1 signaling may inhibit NFATc1 and osteoclast differentiation via upregulation of MafB and/or other NFATc1 inhibitors.

### IL-33 inhibits osteoclastogenesis *in vitro*

**Figure 3:**
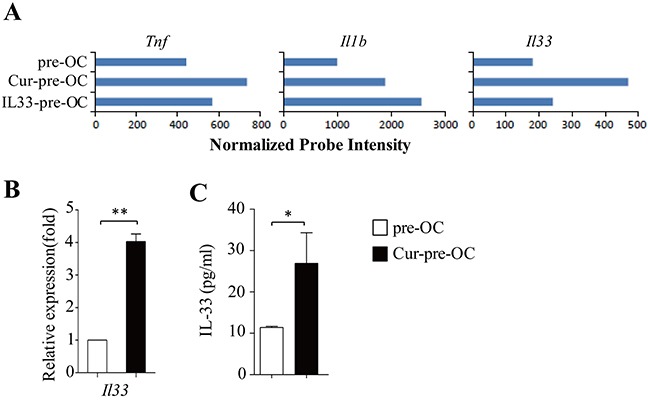
Dectin-1 stimulates IL-33 expression in OC progenitors **(A)** Shown are gene expression levels of the indicated cytokines extracted from microarray data. **(B)** qPCR assessed the expression of *Il33* in pre-OC and Cur-pre-OC. **(C)** ELISA assessed IL-33 secretion in the culture supernatants. Results shown are the mean + SD of at least three **(B,C)** independent experiments. *P < 0.05; **P < 0.01.

Dectin-1 signaling stimulates the production of some inflammatory cytokines [19, 20], which may be involved in osteoclast differentiation. To address this issue, microarray data were examined. Cur-pre-OCs expressed higher levels of *Tnf*, *Il1b* and *Il33* as compared to pre-OCs (Figure 3A). qPCR and ELISA further confirmed the increased expression of IL-33 in Cur-pre-OCs compared to pre-OCs (Figure 3B, 3C). These results demonstrated that dectin-1 activation increased IL-33 expression in osteoclast precursors.

**Figure 4:**
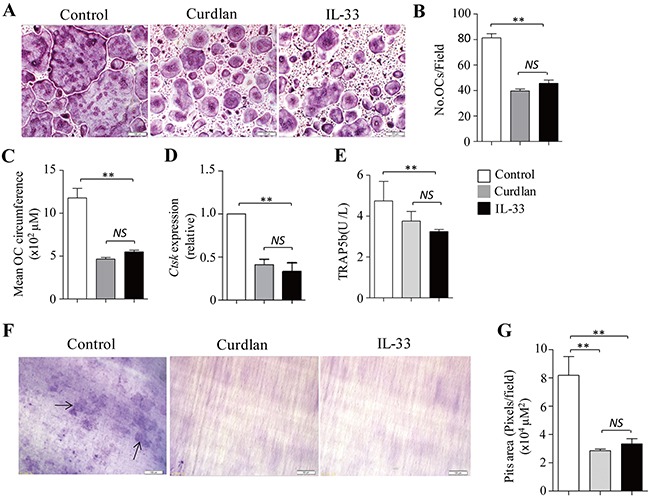
IL-33 inhibits osteoclastogenesis **(A-E)** Mouse BMCs were cultured with M-CSF for 2 days and with M-CSF plus RANKL for 3 days. At day 2, cells were treated with curdlan or IL-33 (30 ng/mL), untreated cells were used as control. **(A)** Shown are representative TRAP^+^ cells from the cultures. **(B)** The number of OCs (> 3 nuclei) as obtained in **(A)**. **(C)** Mean cell circumference of OCs as obtained in **(A)**. **(D)** qPCR assessed *Ctsk* expression in the cells. **(E)** ELISA assessed TRAP5b secretion in the cultures. **(F)** Mouse BMCs were cultured with M-CSF plus RANKL in the presence or absence (control) of curdlan or IL-33 on dentin slices for 7 days. Shown are the resorption pits on dentin slices. **(G)** Summarized results of three independent experiments obtained in **(F)**. Data are presented as mean ± SD of at least three **(B-E,G)** independent experiments. *P < 0.05; **P < 0.01.

TNF-α and IL-1β were shown to promote but not inhibit osteoclast differentiation [21, 22]. We next examined the role of IL-33 in dectin-1-induced inhibition of osteoclast differentiation. Osteoclasts were generated *in vitro* in the presence of M-CSF plus RANKL with or without addition of curdlan or IL-33. IL-33 potently inhibited the development of osteoclasts (Figure 4A) by decreasing the cell number and size of osteoclasts as compared with untreated control (Figure 4B, 4C); while IL-33 induced comparable inhibition on osteoclastogenesis as compared to curdlan (Figure 4A–4C).

By using GEP analyses, we found that osteoclast precursors treated with IL-33 (IL33-pre-OCs) expressed lower levels of *Nfatc1* as compared to pre-OC (Figure 2A). qPCR and Western-blots further confirmed the decreased expression of NFATc1 in IL33-pre-OCs compared to pre-OCs (Figure 2B, 2C). In addition, the expression levels of *Mafb*, *Irf7*, *Irf8* and *Irf9* were increased in IL33-pre-OCs compared to pre-OCs (Figure 2A, 2D). Furthermore, similar to Cur-pre-OCs, IL33-pre-OCs expressed significantly lower levels of *Ctsk* and TRAP5b than pre-OCs (Figure 4D, 4E). Finally, as compared to untreated control, IL-33 inhibited RANKL-induced osteoclast bone resorption (Figure 4F, 4G), at levels comparable to that induced by curdlan treatment (Figure 4F, 4G). These results demonstrated the inhibitory role of IL-33 in osteoclast differentiation, suggesting that dectin-1 signaling may inhibit osteoclastogenesis via upregulation of IL-33.

### Blocking ST2 partially abrogates curdlan-induced inhibition of osteoclastogenesis

**Figure 5:**
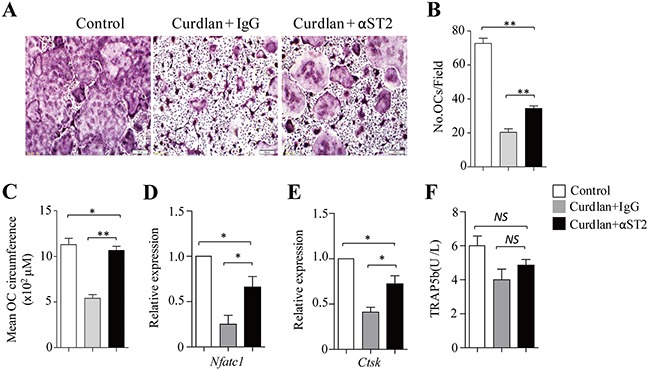
Blocking ST2 partially abrogates dectin-1-induced inhibition of osteoclastogenesis **(A)** Mouse BMCs cells were cultured with M-CSF for 2 days and with M-CSF plus RANKL for 3 days. At day 2, cells were treated with or without (control) curdlan in the presence of a ST2 blocking antibody (αST2) or a control IgG (IgG). Cells were stained for TRAP^+^ cells. (**B** and **C**) The cell number **(B)** and mean cell circumference **(C)** of OCs as obtained in **(A)**. (**D** and **E**) qPCR assessed *Nfatc1* **(D)** and *Ctsk* **(E)** expression in the cells as obtained in **(A)**. **(F)** ELISA assessed TRAP5b secretion in the cultures. Data are representative of three **(A)** independent experiments or presented as mean ± SD of three **(B-F)** independent experiments. *P < 0.05; **P < 0.01.

To examine the role of IL-33 in dectin-1-induced inhibition of osteoclastogenesis, a ST2 (the IL-33 receptor) blocking antibody (αST2) was used during osteoclast culture. The addition of αST2 compared to control IgG increased the generation of osteoclasts in curdlan-treated cultures (Figure 5A), as demonstrated by significantly higher osteoclast cell number and size in the cultures treated with curdlan plus αST2 compared to curdlan alone (Figure 5B, 5C), while lower cell number and size of osteoclasts were obtained in the cultures treated with curdlan plus αST2 compared to untreated controls (Figure 5A–5C), indicating that blocking ST2 partially abrogated curdlan-induced inhibition of osteoclast differentiation. Though as compared to untreated cells, cells treated with Curdlan plus αST2 expressed lower levels of *Nfatc1* and *Ctsk* (Figure 5D, 5E), these cells expressed higher levels of *Nfatc1* and *Ctsk* than cells treated with curdlan alone (Figure 5D, 5E). Furthermore, cells treated with Curdlan plus αST2 slightly increased the expression of TRAP5b as compared to curdlan-treated cells (Figure 5F). Collectively, these results demonstrated the important role of IL-33 in mediating dectin-1-induced inhibition of osteoclastogenesis.

## DISCUSSION

Abnormal osteoclast activation is a major cause of osteolytic bone diseases (OBDs); therefore, targeting osteoclasts may have important clinical significance in the therapy of OBDs [3, 6, 7, 23]. In this study, we found that dectin-1 activation with curdlan inhibited RANKL-induced osteoclast differentiation by reducing osteoclast cell number and size *in vitro*. Dectin-1 activation also decreased the expression of Dcstamp, the key regulator of osteoclast precursor differentiation and fusion, and TRAP and Cathepsin K, which are essential for osteoclastic bone resorption. In addition, in the functional tests, we found that dectin-1 activation inhibited bone resorption of RANKL-induced osteoclasts. These results are consistent with previous observations that intravenous injection with *Candida albicans* enhanced new bone formation in mice [24], and dectin-1 activation in osteoclast precursors inhibited RANKL-induced osteoclast differentiation *in vitro* [18]. Furthermore, curdlan treatment failed to suppress dectin-1^−/−^ osteoclast differentiation. Thus our data demonstrated that dectin-1 activation inhibits osteoclast differentiation and function.

We and others found that dectin-1 activation inhibited NFATc1 expression in osteoclast precursors [18]. However, the dectin-1 downstream signals responsible for NFATc1 inhibition were not fully defined. In this study, we found that dectin-1 activation enhanced the expression of transcription factors MafB, Bcl6, IRF7, IRF8 and IRF9. MafB and Bcl6 are known inhibitors of NFATc1 and osteoclast differentiaton [12]. The IRF family member IRF8 was also reported to inhibit NFATc1 and osteoclast differentiaton [12]. These data suggested that dectin-1 signaling may inhibit NFATc1 expression through upregulation of MafB, Bcl6 and IRF8. In contrast, Yamasaki et al. reported that dectin-1 inhibited NFATc1 expression through the inhibition of syk/c-fos downstream signaling [18]. Thus, our data reveals new insights into dectin-1-induced inhibition of NFATc1 expression and osteoclast differentiation.

It was reported that dectin-1 stimulates macrophages to produce some pro-inflammatory cytokines, such as TNF-α, IL-6 and IL-1β [19, 20]. However, these factors are related to the stimulation of osteoclast differentiation [21, 22, 25, 26]. In this study, we identified IL-33 as a new cytokine that was upregulated by dectin-1 and was related to the inhibition of osteoclast differentiation. Functional tests showed that addition of IL-33 inhibited osteoclast precursors to differentiate into mature osteoclasts and reduced their bone resorptive activity. This result is consistent with previous observations that IL-33 inhibits osteoclastogenesis [27, 28]. In addition, blocking IL-33/ST2 by using a ST2 blocking antibody partially abrogated dectin-1 induced inhibition of osteoclastogenesis. Mechanistic studies revealed that IL-33 increased MafB, IRF7, IRF8 and IRF9 and decreased NFATc1 expression in osteoclast precursors. And blocking ST2 increased NFATc1 expression in dectin-1-activated osteoclast precursors. Thus, we identify IL-33 as an important mediator for dectin-1-induced inhibition of osteoclastogenesis.

Notably, the concentrations of IL-33 in supernatants of curdlan-treated osteoclast precursors were much lower than those we used to efficiently inhibit osteoclast differentiation *in vitro*. The reasons for this discrepancy are unclear. IL-33 is a nuclear cytokine, which is released via cell necrosis. Full length IL-33 can be cleaved by a wide range of proteases, such as caspase-1, elastase, chymase and tryptase, leading to production of different IL-33 variants [29–32]. Full length IL-33 and its cleaved variants may all exhibit some bioactivities but with different intensities [29–32]. Therefore, we first suggested that the bioactivity of the natural IL-33 produced by curdlan-treated osteoclast precursors might be much higher than the commercial synthetic IL-33 that we purchased from the company. Second, after release, most of IL-33 might be captured by the adjacent cells and only little amount of IL-33 was released into the supernatant. Third, the IL-33 ELISA kit may detect only some of IL-33 variants.

In summary, our study demonstrates that dectin-1 activation potently inhibits osteoclast differentiation and bone resorption function. Dectin-1 activation increases MafB and decreases NFATc1 expression. Dectin-1 activation increases the expression of IL-33, which is an important mediator for dectin-1-induced inhibition of osteoclast differentiation and bone resorptive function. Our study has provided new insights into the mechanisms of dectin-1-induced inhibition of osteoclastogenesis and may provide new targets for the therapy of OBDs.

## MATERIALS AND METHODS

### Mice

Balb/c mice were purchased from the Jackson Laboratory. Mice were bred and maintained in pathogen-free facilities at the First Hospital Animal Center of Jilin University. 6-8 weeks old mice were used for experiments. All animal experimental procedures were reviewed and approved by the Animal Ethical Committee of First Hospital of Jilin University.

### Reagents

Recombinant mouse M-CSF (315-02), RANKL (315-11) and IL-33 (210-33) were purchased from Peprotech. Mouse ST2/IL-1R4 neutralization antibody (AF1004) were purchased from R&D Systems. Curdlan (C7821) was purchased from Sigma-Aldrich.

### Osteoclast generation *in vitro*

Bone marrow cells (BMCs)-derived osteoclasts were generated as described previously [33]. In brief, cells were cultured in α-minimum essential medium (α-MEM; GIBCO) supplemented with FBS (10%), L-glutamine (2 mM) and 100 U/mL penicillin (Invitrogen) and 100 mg/mL streptomycin (Invitrogen). BMCs were cultured with M-CSF (10 ng/mL) for 2 days. At day 2 and 4, culture medium was removed and replaced with fresh medium containing M-CSF (10 ng/mL) and RNAKL (10 ng/mL). At day 2 and 4, some cultures were added with M-CSF/ RNAKL plus Curdlan (10 μg/mL unless otherwise indicated) or IL-33 (50 ng/mL). At day 5, cells were processed for Tartrate-resistant acid phosphatase (TRAP) staining, or collected for gene expression analysis by qPCR. Cytokines in culture supernatants were assessed by ELISA.

In blocking experiments, BMCs were cultured with M-CSF (10 ng/mL) for 2 days. At day 2 and 4, cells were cultured with M-CSF/ RNAKL with or without addition of curdlan in the presence of a ST2 neutralization antibody (αST2) (5 μg/mL) or control IgG (5 μg/mL). At day 5, cells were processed for TRAP staining, or cells and culture supernatants were collected for gene expression by qPCR or ELISA.

### Tartrate-resistant acid phosphatase (TRAP) staining

BMCs were cultured with M-CSF for 2 days and with MCSF plus RANKL for 3 days. In some cultures, cells were treated with curdlan (10 μg/mL) or IL-33 (50 ng/mL) at day 2 and day 4. In ST2 blocking experiments, cultures were treated with curdlan in the presence of αST2 (5 μg/mL) or a control IgG (5 μg/mL) at day 2 and day 4. At day 5, culture medium was removed and cells were fixed and stained with Acid Phosphatase, Leukocyte (TRAP) Kit (Sigma) according to the manufacturer’s instructions. TRAP^+^ cells with more than three nuclei were considered as osteoclasts. Osteoclast circumference was calculated by the formula: 3.14 × (mean diameter).

### Real time-polymerase chain reaction

qPCR was performed as previously described [34]. Total RNA was extracted from cells by using an RNeasy Mini kit (Qiagen) according to the manufacturer’s instructions. Primer sets used for these analyses are: *Il33*, 5′-TTC CAA CTC CAA GAT TTC CC and 5′-TGT CAA CAG ACG CAG CAA A; *Dcstamp*, 5′-CCC GCT GAA TAA GAA GGA AA and 5′-ATG GAG GAG ATG AGC CGA TA; *Ctsk*, 5′-ACG GAG GCA TTG ACT CTG AAG ATG and 5′-GGA AGC ACC AAC GAG AGG AGA AAT; *Nfatc1*, 5′-GCC TTT TGC GAG CAG TAT CT and 5′-TAT GGA CCA GAA TGT GAC GG; and *Gapdh*, 5′-TGC ACC ACC AAC TGC TTA GC and 5′-GGA TGC AGG GAT GAT GTT CT.

### Enzyme-linked immunosorbent assay (ELISA) and western blot analyses

IL-33 and TRAP5b ELISA kits were purchased from R&D Systems and Elabscience Biotechnology Co., Ltd, respectively. ELISA assays were performed according the manufacturer’s instructions.

Western blot assay was performed as previously described [34]. Anti-mouse NFATc1 and β-actin antibodies were purchased from Cell Signaling Technology (CST).

### Gene-expression profiling

BMCs were cultured with M-CSF for 2 days. At day 2, culture medium was removed and replaced with fresh medium containing M-CSF/RNAKL (10ng/ml) with or without addition of curdlan (10 μg/mL) or IL-33 (50 ng/mL). At day 4, cells were collected and stored in Trizol reagent (Invitrogen) at −80°C. Samples were sent to OneArray (http://www.OneArray.com.cn/, Beijing, China) for transcription profiling via genome-wide microarrays, and the subsequent data analysis was also performed by OneArray.

### Bone resorption assay

For the bone resorption assay, dentin slices (IDS) were soaked with culture medium for 2h in 96-well plate, BMCs were cultured for 7 days with M-CSF (10 ng/mL) and RANKL (10 ng/mL) in the presence or absence of curdlan on the dentin slices. Cells on dentin slices were removed by 5% sodium hypochlorite, and pits were stained with 1% toluidine blue. The resorption pits were visualized by light microscopy.

### Statistical analysis

The Student *t* test (2 groups) and ANOVA (> 2 groups) were used to compare various experimental groups. A P value of less than 0.05 was considered significant.
